# Assessment of Dietary Intake and Eating Attitudes in Recreational and Competitive Adolescent Rock Climbers: A Pilot Study

**DOI:** 10.3389/fnut.2019.00064

**Published:** 2019-05-10

**Authors:** Marisa K. Michael, Lanae Joubert, Oliver C. Witard

**Affiliations:** ^1^Department of Health Sciences and Sport, University of Stirling, Stirling, United Kingdom; ^2^School of Health and Human Performance, Northern Michigan University, Marquette, MI, United States

**Keywords:** eating attitudes, dietary recall, lowenergy availability, climbing ability, EAT-26, disordered eating, youth, nutrition

## Abstract

The dietary intake and eating attitudes of adolescent climbers has not previously been studied. To fill this knowledge gap, we administered three surveys to 22 rock climbers (13 males, 9 females, age 14.2 ± 1.9 years): climbing ability, three-day dietary recall, and Eating Attitude Test-26 (EAT-26). The majority (82%) of climbers did not meet their target energy intake (target = 2,471 ± 493 kcal·day^−1^; actual = 1,963 ± 581 kcal·day^−1^) (*p* = 0.003) and 86% of climbers consumed below their target carbohydrate intake (target = 283 ± 67 g·day^−1^; actual intake = 226 ± 72 g·day^−1^) (*p* = 0.009). Average dietary protein intake was 95 ± 51 g·day^−1^, with the majority of climbers meeting their target intake of 88 ± 21 g (*p* = 0.580). Seventy-three percent of climbers consumed below their target dietary fat intake (target = 90 ± 21 g·day^−1^; actual = 69 ± 20 g·day^−1^) (*p* = 0.001). Average EAT-26 scores were 5.3 ± 4.1, indicating minimal risk of disordered eating attitudes/behaviors. There were no significant differences in boulderers vs. top rope climbers for energy/macronutrient intake, BMI, and EAT-26 score. There were no associations between energy intake and EAT-26 score (*R*^2^ = 0.245, *p* = 0.271) or climbing ability and EAT-26 score (*R*^2^ = *p* = 0.217). These data suggest that, with the exception of dietary protein intake, adolescent climbers fail to meet target dietary intakes, and exhibit minimal risk of disordered eating.

## Introduction

Rock climbing is an increasingly popular sport, gaining worldwide attention as a new event in the Tokyo 2020 Olympics. The physiological demands of climbing include upper body strength, muscular endurance, flexibility, and a reliance on both aerobic and anaerobic energy systems (1–3). Elite climbers are typically shorter and lighter than the general population (2, 4), suggesting that climbing success depends on a high strength-to-mass ratio. Accordingly, dietary patterns must be carefully considered to ensure optimal body composition while providing sufficient energy to avoid low energy availability/relative energy deficiency in sport (RED-S). RED-S refers to inadequate energy intake to support various body functions and optimal performance (5). In 2018, the International Olympic Committee updated this definition, which includes the concept of low energy availability (LEA). Operationally, energy availability (EA) is calculated as Energy Intake (EI) (kcal)—Exercise Energy Expenditure (EEE) (kcal)/Fat Free Mass (FFM) (kg) where exercise energy expenditure (EEE) is calculated as the additional energy expended above that of daily living during the exercise bout. The overall result is expressed relative to FFM, reflecting the body's most metabolically active tissues” (6).

There is anecdotal evidence that some climbers exhibit disordered eating behaviors in order to minimize body weight and thus potentially enhance performance. A status of low energy availability may result, which can compromise health and performance (5). On this basis, the Austrian Sportclimbing Organization has banned climbers with a BMI of <18 (males) and <17 (females) from competing in an effort to prevent disordered eating, and concern has been raised regarding the risk of adolescent climbers developing anorexia athletica (7).

Several studies have evaluated the energy expenditure of adult (2, 3, 8) and adolescent (9) climbers. However, only two small-scale studies have assessed dietary intakes in adult climbers (10, 11). In this regard, Merrells et al. profiled the dietary patterns of two male rock climbers during a 5-week outdoor rock climbing trip, estimating these climbers to be in a 40% energy deficit. Both climbers lost weight, equivalent to 5.8 and 16.1% of their initial body weight over this 5 week period. Moreover, Zapf et al. (11) administered 7-day weighed food diaries to analyze dietary patterns in 20 elite climbers, reporting that ~50% failed to meet macronutrient intake recommendations. To our knowledge, no studies have assessed dietary intake patterns and risk of disordered eating in adolescent climbers. This gap in knowledge is relevant given that adolescent climbers in a state of RED-S may suffer from decreased growth and maturation status (12, 13).

The development of eating disorders in young athletes typically begins between ages 14 and 17 years (14). Competitive athletes from aesthetic and weight-class sports often exhibit extreme dieting behaviors that may compromise health and performance (15). In this regard, estimated prevalence rates of disordered eating in male athletes is 0–19% and 6–45% in female athletes (16). Therefore, the aim of this pilot study was to assess dietary intake and eating attitudes associated with disordered eating among recreational and competitive adolescent climbers aged 11–17 years. Moreover, we explored the relationship between dietary intake, body weight, climbing ability, and risk for disordered eating in our cohort of adolescent rock climbers. We hypothesized that the majority of climbers would fail to meet target energy and macronutrient intakes and that eating attitudes associated with disordered eating would be highly prevalent in adolescent rock climbers.

## Methods

### Participants

A pilot study cohort of 22 rock climbers (13 males, 9 females) from two climbing gymnasiums volunteered to participate in this study. The researcher was a registered dietitian who met with climbers and their coaches to verbally explain the purpose of the research. The coaches approved the administration of three surveys during scheduled team practice. Prior to participating in the study, both the parent/guardian of the participants and the participants themselves signed a written consent form allowing participation in the study. Consent was both written and informed. This study was approved by the University of Stirling General University Ethics Panel.

### Surveys

Each participant completed four assessment surveys: health history, climbing ability, dietary recall, and eating attitudes, all under the supervision of the same registered dietitian to ensure accuracy and precision in responses. Climbing coaches were present during survey completion only to help participants determine climbing ability.

#### Health History Survey

A health history survey was administered to assess whether any climbers had medical conditions or medication/supplement use that would alter their food choices. This survey contained questions regarding the presence of current medical diagnoses that may have impacted food intake choices, such as celiac disease, diabetes, or eating disorders. This also included questions regarding weight, height, gender, and age. This information was used to determine Body Mass Index information based on Center for Disease Control guidelines.

#### Climbing Ability Survey

The climbing ability survey contained questions from the International Rock Climbing Research Association (IRCRA) recommendations to create consistency within rock climbing research. We assessed years of climbing experience, number of climbing competitions completed in past year, hours per week of training, whether the climber identified as primarily a boulderer or top rope climber, and most difficult route climbed for both bouldering and top rope with this survey. Route difficulty was defined as highest difficulty obtained by current best redpoint (which is defined as 3–5 attempts to complete a route—this allows practicing a route before attempting to complete it, rather than trying to complete it within one attempt). Climbing coaches assisted participants in determining the correct climbing ability score. Ratings were converted from Vermin rating system (bouldering) and Yosemite Decimal System (top rope) to the IRCRA Reporting Scale (17), which distinguishes five climbing ability levels: lower grade, intermediate, advanced, elite, higher elite.

#### Dietary Recall

A 3-day food recall was administered to assess habitual dietary intake for normal weekdays to mimic usual intake, since training sessions take place after school, thereby providing a “snapshot” of climbers' intakes on a usual training day. These data enabled us to assess whether the climbers were eating enough to meet estimated energy and macronutrient needs to support their training for that day. Climbers reported their dietary intake for all meals, snacks, and beverages excluding water for the preceding 3 days. The registered dietitian gathered responses via an on-site interview with verbal prompts to ensure complete responses, and to clarify portion sizes, types of foods, brand names, and cooking methods in order to achieve accurate responses. Diet was analyzed using Nutritics Professional Edition software (version 4.315). When exact foods were not located in the database, similar foods were selected that represented the macronutrient content of the food reported on the dietary recall form and during the dietary recall interview.

#### Eating Attitude Test Survey

Risk of disordered eating was assessed using the Eating Attitude Test-26 survey (EAT-26) (18) in accordance with published scoring guidelines and with author permission. EAT-26 contains a Likert scale where respondents answer on a scale of “always” to “never” on questions relating to attitude toward food, dieting, and body image. A score of ≥20 or a “yes” answer to five behavioral questions was considered indicative of disordered eating behavior. These questions probed for information regarding the use of binging, vomiting, laxatives, and exercise as a means to control weight. Parents/guardians of any participant that met these criteria were referred to an eating disorder professional for further assessment.

Coaches were only present during administration of the surveys only to assist participants in the correct classification of climbing ability. Participants confidentiality was assured for all other survey results, including health history and EAT-26 responses, in order to ensure honest responses to the EAT-26 survey. Coaches and parents were not made aware of any results, with the exception of parents being notified if a response on the EAT-26 survey indicated need for referral to an eating disorder professional.

### Estimating Energy and Macronutrient Needs

Resting metabolic rate (RMR) was calculated using Nutritics software with the Schofield equation (19) [0.056 × wt (kg) + 2.898]. The Schofield equation was used since it is appropriate for adolescents, and was developed using 10–17-year-olds which closely matches the age of our cohort (19). To estimate the dietary energy needs of adolescent athletes, RMR was multiplied by a physical activity factor of 1.6 for males and 1.5 for females (13). In accordance with published guidelines, target dietary carbohydrate (CHO) was set at 5g·kg^−1^ body mass (BM)·day (12, 13) and target dietary protein was based on an estimate of 1.6g·kgBM^−1^·day^−1^ for young athletes (12). A target for dietary fat intake was set at 20–35% of total energy intake, based on the Sports Dietitians of Australia position statement for adolescent athletes (12). When comparing dietary intake for advanced vs. intermediate/low level climbers, males vs. females, and boulderers vs. top ropers, data are expressed as a percent of target recommendations.

## Statistical Analysis

Statistical analysis was performed using SPSS software (version 22, 2016) and Microsoft Excel for Mac (version 16.11, 2017). An independent samples Students *t*-test was used to analyze differences in variables between groups (e.g., elite/advanced vs. intermediate/low grade climbers). A paired samples Students *t*-test was used to analyze differences in variables within groups (e.g., target vs. actual energy intakes) with significance level set at *p* < 0.05. A Pearson correlation coefficient determined the relationship between energy/macronutrient intake and EAT-26 scores, climbing ability and EAT-26 scores, body weight and EAT-26 scores, BMI and EAT-26 scores, and energy intake and EAT-26 scores. This statistical test was also used to determine the relationship between energy and macronutrient intake and training hours. Correlation values (*R*^2^*)* were set as <0.2: weak correlation, 0.5: medium correlation, and >0.8: strong correlation (20). Data are presented as means ± standard deviation (SD), unless otherwise stated.

## Results

### Participant Demographics

Participant demographics are shown in Table 1. The average BMI for the study cohort was 20.1 ± 2.4. A BMI of ≤17 was reported in four participants, all other participants reported BMI value between 19 and 25. The average weight percentile was 51 ± 26, with 12 climbers ≤50th percentile. The average height percentile was 56 ± 28, with ten climbers ≤50th percentile. Based on the IRCRA scale, average climbing ability was 19.8 ± 3.8 for males and 19.1 ± 3.1 for females.

**Table 1:** Descriptive details of rock climbers.

**Characteristics**	**All climbers**	**Males *n* = 13**	**Females *n* = 9**
Age	14.2 ± 1.9	14.8 ± 1.5	13.3 ± 2.2
Height (cm)	165.0 ± 12.4	171 ± 10.2	156 ± 10.2
Weight (kg)	55.1 ± 13.0	61.1 ± 12.3	46.6 ± 8.5
BMI (kg/m^2^)	20.1 ± 2.4	20.1 ± 2.2	19 ± 2.3
Weight percentile (CDC Growth Charts, USA)	51 ± 26	54.7 ± 26.2	45.6 ± 27.1
Height percentile (CDC Growth Charts, USA)	56 ± 28	56.8 ± 25.9	55.4 ± 34.4
Climbing ability (IRCRA): all climbers	19.6 ± 3.5	19.8 ± 3.8	19.2 ± 3.1
Climbing ability (IRCRA): boulderers (*n* = 10)	20.0 ± 2.9	20.9 ± 3.1	19.4 ± 2.2
Climbing ability (IRCRA): top rope climbers (*n* = 12)	18.0 ± 4.5	18.3 ± 4.6	18.6 ± 4.5
Years in sport	4.6 ± 3.7	4.8 ± 4.3	4.1 ± 2.8
Typical training volume (h/week)	11.1 ± 7.6	11.5 ± 9.2	10.6 ± 5.3
Competitions per year	7.0 ± 5.0	7.1 ± 6.0	6.8 ± 3.4

*Values represent mean ± SD*.

One climber reported being vegan, and another climber reported food allergies. Four climbers reported having asthma but no other climbers reported any other health conditions. None of the participants reported medications and/or dietary supplements that had the potential to substantially impact dietary energy/macronutrient intake nor influence energy expenditure.

There were *n* = 0 lower grade climbers, *n* = 4 intermediate grade climbers, *n* = 15 advanced climbers, *n* = 2 elite climbers, and *n* = 0 higher elite climbers in our cohort according to the IRCRA grading scale. Our cohort had been climbing for an average of 5 ± 4 years, and participated in an average of 7 ± 5 competitions in the past year. Eighteen out of the 22 climbers had participated in competitions within the past year.

### Dietary Intake

#### Energy and Macronutrient Intakes of All Climbers Combined

Dietary intakes of all climbers combined are shown in Table 2. Eighty-two percent of climbers consumed below their target energy dietary intake of 2,471 ± 493 kcal (*p* = 0.002) and 45 ± 4 kcal·kgBM^−1^·day^−1^ (*p* = 0.003). The average daily energy dietary intake was 1,963 ± 581 kcal and 37 ± 11 kcal·kg^−1^·day^−1^, which is ~79% of target energy needs when expressed in absolute terms and 82% of target needs when expressed relative to body mass.

**Table 2:** Dietary intakes of all climbers combined.

**Nutrient**	**Target intake**	**Actual intake**	**Percent of climbers not meeting target intakes**	***p*-value**
**Energy**				
total kcal·day^−1^	2471 ± 493	1963 ± 581	82%	*p* = 0.002
kcal·kg^−1^·day^−1^	45 ± 4	37 ± 11	–	*p* = 0.003
**Carbohydrate**				
total g·day^−1^	283 ± 67	226 ± 72	86%	*p* = 0.009
g·kg^−1^·day^−1^	5.0	4.3 ± 1.6	–	*p* = 0.044
% total intake	>50	49 ± 8	–	–
**Protein**				
total g·day^−1^	88 ± 21	95 ± 51	23%	*p* = 0.584
g·kg^−1^·day^−1^	1.6	1.7 ± 0.8	–	*p* = 0.494
% total intake	15–20	19 ± 5.1	–	–
**Fat**				
total g·day^−1^	90 ± 21	69 ± 20	73%	*p* = 0.001
g·kg^−1^·day^−1^	1.7 ± 0.3	1.3 ± 0.5	–	*p* = 0.003
% total intake	20–35	32 ± 5.1	–	–

*Recommended total energy intake based on the Schofield equation [0.056 × wt (kg) + 2.898] × 1.6 activity factor for males, or 1.5 activity factor for females. Target carbohydrate intake set at 5 g·kgBM^−1^·day^−1^ (13). Recommended dietary protein intake set at 1.6 g·kg^−1^·day^−1^ and recommended percent fat intake based on 20–35% total energy intake (12). Data are expressed as means ± SD. P ≤ 0.05 is considered significant*.

Eight-six percent of climbers consumed below their target CHO needs of 283 ± 67g (*p* = 0.009) and 5.0 g·kgBM^−1^·day^−1^ (*p* = 0.044). Average dietary CHO intake was 226 ± 72 g·day^−1^ and 4.3 ± 1.6 g·kgBM^−1^·day^−1^, which is approximately 79% of target CHO intake when expressed in absolute intake, and 86% of target CHO intake when expressed relative to body mass.

Twenty three percent of climbers consumed below their target protein intake of 88 ± 21 g (*p* = 0.584) and 1.6 g·kgBM^−1^·day^−1^, (*p* = 0.494). Average dietary protein intake was 95 ± 51 g·day^−1^ expressed in absolute intake, and 1.7 ± 0.8 g·kgBM^−1^·day^−1^ when expressed relative to body mass.

Seventy three percent of climbers consumed below their target dietary fat intake of 90 ± 21 g·day^−1^ (*p* = 0.001) and 1.7 ± 0.3 g·kgBM^−1^·day^−1^ (*p* = 0.003). Average fat intake was 69 ± 20 g·day^−1^ when expressed as absolute intake, and 1.3 ± 0.5 g·kgBM^−1^·day^−1^ when expressed relative to body mass.

#### Comparisons of Energy and Macronutrient Intake Between Groups

**Figure 1:**
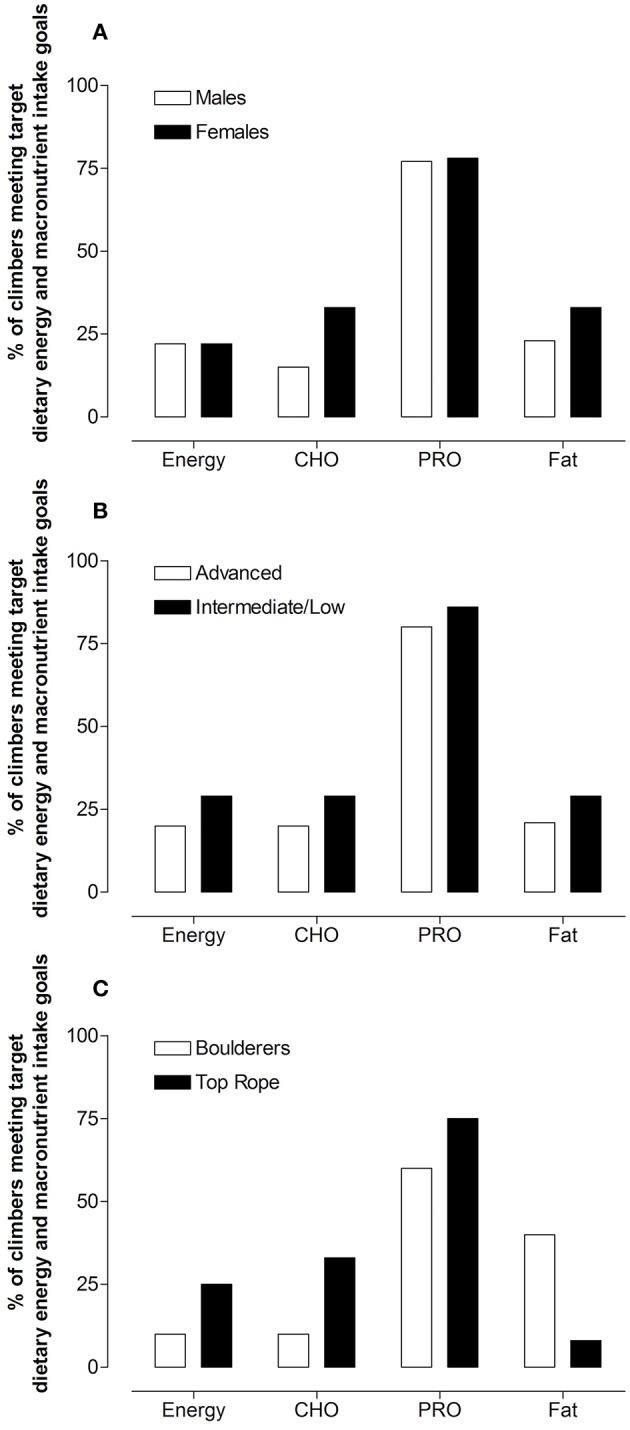
Percentage of males vs. females **(A)**, advanced vs. intermediate/low grade **(B)**, and boulderers vs. top rope **(C)** climbers that met dietary intake targets.

Expressed relative to body mass (g·kgBM^−1^·day^−1^), there were no differences in energy (*p* = 0.335), CHO (*p* = 0.155), protein (*p* = 0.265), or fat intake (*p* = 0.730) between males and females (Table 3). The percentage of males vs. females that met their target energy (22 vs. 22%), CHO (15 vs. 33%), protein (77 vs. 78%), and fat (23 vs. 33%), respectively, are expressed in Figure 1A.

**Table 3:** Dietary intake patterns and eating attitudes in male vs. female rock climbers.

		**Males** ***n*** **=** **13**			**Females** ***n*** **=** **9**		
	**Target**	**Actual**	***p*-values**	**Target**	**Actual**	***p*-values**	**♂ vs. ♀**
Height (cm)	—	171 ± 10.2	—	—	156 ± 8.5	—	*p* = 0.004
Weight (kg)	—	61.1 ± 12.4	—	—	46.6 ± 8.5	—	*p* = 0.004
BMI (kg/m^2^)	—	20.9 ± 2.2	—	—	19 ± 2.3	—	*p* = 0.059
Energy intake (kcal)	2797 ± 357	1963 ± 581	*p* = 0.003	2000 ± 149	2047 ± 649	*p* = 0.308	*p* = 0.335
CHO intake (g·kgBM^−1^·day^−1^)	5.0	4.3 ± 1.6	*p* = 0.003	5.0	3.9 ± 1.5	*p* = 0.625	*p* = 0.155
Protein intake (g·kgBM^−1^·day^−1^)	1.6	1.8 ± 1.0	*p* = 0.413	1.6	1.6 ± 0.3	*p* = 0.663	*p* = 0.265
Fat intake (g·day^−1^)	101 ± 21	71 ± 22	*p* = 0.001	74 ± 6	66 ± 17	*p* = 0.192	*p* = 0.578
EAT-26 score	>20	6.8 ± 4.4	—	>20	3.2 ± 2.8	—	*p* = 0.031

*P ≤ 0.05 is considered significant*.

Elite/advanced climbers consumed more dietary protein (1.8 g·kg^−1^·day^−1^) than intermediate/low grade climbers (1.7 g·kg^−1^·day^−1^, *p* = 0.043), with no statistical differences in energy (*p* = 0.583), CHO (*p* = 222) or fat (*p* = 0.193) intake between climbing ability groups. The percentage of climbers that met target energy or macronutrient targets was similar between elite/advanced and intermediate/low grade climbers (Figure 1B). Figure 1C expresses the percentage of boulderers compared to top rope climbers, respectively, which met target energy (10 vs. 25%), CHO (10 vs. 33%), protein (60 vs. 75%), and fat (40 vs. 8%). Table 4 expresses dietary intakes, EAT-26 scores, body weight, and BMI of elite/advanced vs. intermediate/low grade climbers.

**Table 4:** Dietary intakes, EAT-26 score, body weight, and BMI of elite/advanced vs. intermediate/low grade rock climbers.

	**Elite/advanced climbers**	**Intermediate/low climbers**	***p*-values**
Energy intake	2,000 ± 642	1,867 ± 450	*p* = 0.583
% of climbers meeting energy intake target	20%	29%	
CHO intake (g·kg^−1^BM·day^−1^)	4.3 ± 1.6	3.6 ± 1.5	*p* = 0.223
% of climbers meeting CHO intake target	20%	29%	
Protein intake (g·kg^−1^BM·day^−1^)	1.8 ± 0.8	1.7 ± 1.0	*p* = 0.043
% of climbers meeting protein intake target	73%	88%	
Fat intake (g·kg^−1^BM·day^−1^)	1.6 ± 0.3	1.8 ± 0.2	*p* = 0.193
% of climbers meeting fat intake target	27%	29%	
EAT-26 score	5.6 ± 4.7	4.7 ± 2.3	*p* = 0.565
Body weight (kg)	54.4 ± 11.1	56.7 ± 17.1	*p* = 0.752
BMI (kg/m^2^)	19.8 ± 2.2	20.7 ± 2.8	*p* = 0.114

*Numbers expressed as means ± SD. Advanced climbers: males n = 8; females n = 7. Intermediate/low climbers: males n = 5; females n = 2. P ≤ 0.05 is considered significant*.

### Eating Attitudes and Behaviors

An EAT-26 score of ≥20 or a “yes” answer to key behavioral questions is considered a score significant enough to indicate higher risk for disordered eating (18). The average EAT-26 score was 5.3 ± 4.1, indicating an overall low risk of eating attitudes associated with disordered eating patterns. Only 1 participant met the criteria for an eating attitude associated with disordered eating by responding “yes” to a behavioral question. The prevalence of disordered eating attitudes was 4.5%. Although, with one exception, EAT-26 scores did not indicate disordered eating, the average EAT-26 score was higher in males (6.8 ± 4.3) than females (5.3 ± 4.1, *p* = 0.031). Although a statistical difference in EAT-26 scores existed between males and females, these scores were not high enough to indicate high risk for disordered eating. No differences in EAT-26 score were observed between elite/advanced (5.6 ± 4.7) and intermediate/low grade (4.7 ± 2.3, *p* = 0.566) climbers or top rope (5.2 ± 3.9) and boulderers (5.5 ± 4.6, *p* = 0.859). There were no associations between energy/macronutrient intakes and EAT-26 scores (*R*^2^ = 0.245, *p* = 0.271) or climbing ability and EAT-26 scores (*R*^2^ = 0.274, *p* = 0.217) when assessed for all climbers combined. Weak correlations were observed between body weight and EAT-26 scores (*R*^2^ = 0.389, *p* = 0.73) and between BMI and EAT-26 scores (*R*^2^ = 0.415, *p* = 0.55). These data suggest that energy/macronutrient intake, risk for disordered eating patterns, body weight, and BMI have little to no relationship on climbing ability in this cohort. However, the average weight percentile (50.95 ± 26.34) and the average height percentile (56.27 ± 28.92) suggest that these participants are of a general average weight and height, and no participant within the cohort was exceptionally under- or over-weight. Climbing ability was more associated with years the participant had been climbing (*R*^2^ = 0.585, *p* = 0.004) and hours of training per week (*R*^2^ = 0.556, *p* = 0.009).

### Training Hours and Energy and Macronutrient Intake

Weak correlations were observed between training hours and energy intake (*R*^2^ = 0.120, *p* = 0.605), training hours and CHO intake (*R*^2^ = 0.116, *p* = 0.607), training hours and protein intake (*R*^2^ = 0.157, *p* = 0.498) and training hours and fat intake (*R*^2^ = 0.170, *p* = 0.462).

## Discussion

The primary aim of this study was to examine the dietary intakes of adolescent climbers on typical climbing days, and determine the prevalence of disordered eating attitudes in this population. We compared actual dietary intake to estimated target energy and macronutrient needs, and the prevalence of disordered eating attitudes between male and female climbers, elite/advanced and intermediate/low grade climbers and boulderers vs. top rope climbers. Our pilot data suggest that these adolescent climbers were in an energy deficit (with 82% not meeting target needs). Moreover, the majority of climbers failed to meet target dietary CHO (with 86% of climbers not meeting target intake) and fat needs (with 73% of climbers not meeting target intake), while the majority (77%) of climbers met or exceeded target dietary protein needs. When comparing dietary energy and macronutrient intakes between groups, we observed only minimal differences between males and females, advanced and intermediate/low grade climbers, and boulderers and top rope climbers. Finally, we report a general lack of differences in energy/macronutrient intake as well as EAT-26 scores among males vs. females and also among elite/advanced vs. intermediate/low climbers. This observation is not consistent with the previous findings of Sundgot-Borgen and Torstveit (15), which reported greater prevalence rates of disordered eating among elite athletes, and Sundgot-Borgen and Garthe (21), which reported greater disordered eating among females.

Youth climbers commonly exhibit an ectomorph body shape, characterized by a small frame, light build, and moderate volume of lean muscle (8, 22). The energy expenditure of youth climbers (age 10.9 ± 1.7 years) has been compared to children's energy expenditure for stair climbing and easy jogging (9). Youth climbers in our cohort spent an average of 11 hours per week training with a minimum of four hours per week training. Taken together, these data suggest it is plausible that target energy intakes of climbers exceed that of sedentary peers.

It is unknown if climbers intentionally or inadvertently consume less energy when they are in a state of low energy availability. This knowledge is particularly relevant for adolescent climbers who are still growing and may experience negative health implications associated with RED-S. An energy deficit can lead to negative health consequences such as a decrease in bone mineral density, suppression of immune function, and reproductive complications (6). In this regard, Zapf et al. (11) demonstrated that 40% of elite adult climbers failed to meet estimated target energy needs, a finding replicated by Merrells et al. (10), although in this study, the sample size was small (*n* = 2) and the climbers did not have access to normal food stores or preparation methods. Larger-scale free-living observational studies are warranted to describe the typical dietary patterns of both adult and adolescent climbers.

While self-reported values for body weight and calculated BMI appear appropriate in our cohort of climbers based on age and height, our data do not reveal whether climbers were undergoing other physiological adaptations or were classified as in a state of low energy availability. This study limitation may be addressed in future research by tracking growth, body composition, energy intake, bone density, menstrual status (females), and climbing performance in adolescent climbers over a longer time period. Overall, the majority of rock climbers in this study cohort failed to meet target dietary energy, CHO, and fat intakes, expressed in both absolute and relative terms. This preliminary observation is of direct concern to athletes and coaches in rock climbing and further investigation is warranted in larger cohort studies to substantiate these findings.

Typical training sessions for youth climbers may consist of periods of active climbing followed by rest periods on the ground, as well as conditioning such as planks, pull-ups, and sit-ups. Active climbing can also include resting on the wall during a route. Due to the intermittent nature of a climbing session, target CHO needs are likely lower in climbers than endurance or field-based athletes where activity is more continuous. However, adequate CHO intake remains an important consideration for competitive climbers (23) who utilize both aerobic and anaerobic energy systems, and employ explosive movements to complete a route. In this pilot study, we revealed no relationship between energy or macronutrient intake and training hours performed. The climbers that reported greater training hours did not increase their energy intake to compensate for this higher training load, suggesting the risk of RED-S increases with increased training hours. Taken together, these data provide preliminary evidence that adolescent climbers may be at risk of RED-S and thus energy intake of adolescent climbers should be carefully monitored.

The actual dietary protein intake of this cohort of rock climbers generally met or exceeded protein targets. The physiological utilization of dietary protein is particularly crucial for growth (13, 24). However, currently there is a lack of consensus with regards to protein recommendations specifically tailored for adolescent athletes (12). As an indicator of protein requirements, a previous study in youth sprint athletes (ages 12–18) demonstrated that, to achieve positive nitrogen balance, dietary protein intake needed to be between 1.35 and 1.46 g/kg/day for both males and females (25). This level of protein intake was sufficient to support growth. In our study cohort, dietary protein intake averaged 1.6 g/kg/day.

Therefore, based on protein guidelines for athletic young adults (1.3–1.8 g·kgBM^−1^·day^−1^) (12), we assumed that a target protein intake of 1.6 g·kgBM^−1^·day^−1^ was appropriate for adolescent climbers with a normal training volume of ~11 h/week. Based on this criteria, our pilot data suggest that climbers may unintentionally adopt a high protein energy-restricted diet (21). Indeed, studies have reported a loss of fat mass congruent with a maintenance of lean mass in response to an energy restricted high protein diet (26), likely mediated by maintaining rates of muscle protein synthesis despite energy deficit (27). Whereas it appeared that adolescent climbers in this pilot study adopted a high protein, energy restricted diet, it was not possible to discern whether this practice was intentional or by chance. High protein diets have been linked with concerns regarding kidney function and bone loss in clinical populations (i.e. patients with kidney failure) (28). However, these concerns do not apply to athletic populations (29), as recruited in the present study.

Based on anecdotal evidence, adolescent climbers are prone to implementing extreme dieting behaviors in a drive to attain a slim physique in order to enhance performance. Since the body physique of successful climbers is often characterized as light and lean, we anticipated a high prevalence of disordered eating in adolescent climbers with prevalence rates similar to those reported in the literature for aesthetic and weight-class sports. Refuting this hypothesis, the prevalence of attitudes and behaviors associated with disordered eating was ~5%, which is considerably lower than prevalence estimates of 40–42% in aesthetic sports (i.e., gymnastics, figure skating) and 30–35% in weight class sports (i.e., wrestling, boxing) (21). Future larger-scale studies are warranted to comprehensively determine the prevalence of disordered eating in adolescent climbers.

The strengths of our descriptive pilot study include its novelty, the elite/advanced ability of the climbers, 3-day diet data, and use of a validated tool to assess disordered eating behavior risk. However, several limitations must be acknowledged. First, although anthropometric data were self-reported, previous work demonstrated that adolescents report accurate values for height and weight (30). The average height (165 cm), weight (55.1 kg), and BMI (20.1) of our study cohort closely matched the previous control findings of Watts (22) in athletic adolescents (mean height: 167 cm, mean weight: 54.1 kg). However, our cohort was heavier and taller than the climbers presented in Watts (2003). This observation may reflect historical changes in the anthropometrics of adolescent populations over the past 16 years, or the evolving body type of climbers. The sport of climbing has changed, and new research is warranted to determine the anthropometrics of the modern climber.

Second, we acknowledge that self-reported dietary recalls introduce the possibility of under/over-reporting food intake In general, under-reporting in children is less likely in 24-h dietary recalls than in self-reporting surveys in which participants are asked to record their own food intake (31). However, it is difficult to determine whether under-reporting is a result of the misreporting of the kinds and amounts of food consumed, or from other factors influencing the participants' actual food intake.

By 12 years of age, a child's ability to self-report dietary intake is adequate, and over-reporting occurs with multiple-day 24-h recall in approximately 11% of studies using this method (32). Moreover, Rockett et al. (33) demonstrated that a food frequency questionnaire was similar to a dietary recall and could provide accurate information in adolescents. Although the 3-day dietary recall method has its limitations, it is widely used in nutrition research (34). In the present study, there is evidence that adolescent climbers did not misreport their dietary intake given that energy, CHO and fat intakes were systematically lower than target intakes, whereas dietary protein intakes met target recommendations. We assume that diet reporting was accurate with the dietitian present to conduct the dietary interview. Hence, we are confident that dietary intake data collected in this study of adolescent rock climbers was valid. Self-reported climbing ability may vary from actual climbing ability. Self-reported climbing ability has previously been validated as an accurate representation of actual climbing ability in ~25-year-old adults (35). This validation study has not been replicated in adolescents. In the present study, coaches were present during data collection to validate climbing ability, which may have contributed to accurate reporting of climbing ability.

## Conclusion

This descriptive pilot study of dietary intake in adolescent rock climbers suggests that, with the exception of dietary protein, actual energy and macronutrient intakes failed to meet target recommendations. There were no significant differences in dietary intake between males and females and between climbing abilities. This cohort of adolescent rock climbers reported a minimal risk for disordered eating patterns. However, in terms of practical implications, caution should be exercised in this at-risk population since a chronic period of energy deficit may lead to deleterious health and/or performance consequences for adolescent rock climbers. Larger-scale follow-up studies are warranted that assess dietary patterns and risk of disordered eating in the fast growing sport of rock climbing.

## Ethics Statement

This study was carried out in accordance with the recommendations of the General University Ethics Panel of the University of Stirling in Scotland, United Kingdom with written informed consent from all subjects and their parents. The protocol was approved by the General University Ethics Panel. Since the study was conducted with adolescent participants, the primary researcher also completed a background check and obtained approval from local government authorities to interact with minors.

## Author Contributions

MM designed the study, collected, analyzed, and interpreted the data, and drafted the manuscript. OW designed the study, analyzed, and interpreted the data, and edited the manuscript. LJ designed the study and edited the manuscript. All authors gave final approval on the manuscript.

### Conflict of Interest Statement

The authors declare that the research was conducted in the absence of any commercial or financial relationships that could be construed as a potential conflict of interest.
